# Mr. Swift's Cases

**Published:** 1801-06

**Authors:** J. S. Swift

**Affiliations:** Surgeon. Seaflower, Jersey


### *To the Editors of the Medical and Physical Journal.*

Gentlemen,

I Have ſent you a caſe of an inveterate Eruption, occaſioned by taking a "Quack Medicine," called Jeſuit's Drops, for the lues venerea; and a caſe of Cardialgia, cured by the application of flannel; which, if you think worthy of a page in your Journal, you will oblige me by inſerting them.

*Seaflower, Jerſey,* I am, &c.

*March* 16, 1801. J. S. SWIFT, Surgeon.

---

Henry Kelly, belonging to his Majeſty's brig Seaflower, aged 42, of a robuſt habit of body, applied to me, Feb. 23, ult. for a univerſal ſcaly eruption, very red and itching, ſome the ſize of a crown piece. Upon enquiry, he confeſſed having taken a bottle of Jeſuit's Drops, in December laſt, for the venereal diſeaſe; it firſt broke out about his ſcrotum and anus, a few days after he had finiſhed the bottle; the only marks of the diſeaſe remaining were two chancres recently healed. He was diſcharged to duty March 10th inſt. cured by the following treatment: ℞. G. camph. ʒj. ant. tart. op. a. ℈ſs. mucil. q. s. f. pil. xx. omne nocte ſumendus. ℞. Ung. ceræ. ℥ij. hydr. nitrat. rub. g. camph. a. ʒj. M. applic. parti. bis die. Purgatives gatives were taken occafionally; the pills caufed a flight naufea a few nights. 26th, I added a drachm of opium to the ointment, to allay the itching, which was very great when in bed. March 1ft, Complained of great weaknefs, particularly in his knees: ℞. Hauft. cort. Peruv. quotidie.

Thomas Williams for a long time was afflicted with cardialgia, had taken magn. alb. cret. kali, &c. without much benefit. Having known diarrhœas and colics brought on by only leaving off a cloth waiftcoat and putting on a linen one, it ftruck me very probably it was occafioned by a deficiency of external warmth, I therefore applied a double piece of flannel upon his ftomach, and had the pleafure to find him the day following much relieved. In the fpace of fix days he was totally free from the leaft fymptom of his complaint, and has continued fo ever fince, which is nearly three weeks.

